# Deep Learning Combined with Hyperspectral Imaging Technology for Variety Discrimination of *Fritillaria thunbergii*

**DOI:** 10.3390/molecules27186042

**Published:** 2022-09-16

**Authors:** Muhammad Hilal Kabir, Mahamed Lamine Guindo, Rongqin Chen, Fei Liu, Xinmeng Luo, Wenwen Kong

**Affiliations:** 1 College of Biosystems Engineering and Food Science, Zhejiang University, 866 Yuhangtang Road, Hangzhou 310058, China; 2 Department of Agricultural and Bio-Resource Engineering, Abubakar Tafawa Balewa University, Bauchi PMB 0248, Nigeria; 3 College of Mathematics and Computer Science, Zhejiang A & F University, Hangzhou 311300, China

**Keywords:** convolutional neural network, flavonoids, essential oils, saponins, alkaloids, traditional Chinese herbal medicine, *Fritillaria thunbergii*

## Abstract

Traditional Chinese herbal medicine (TCHM) plays an essential role in the international pharmaceutical industry due to its rich resources and unique curative properties. The flowers, stems, and leaves of Fritillaria contain a wide range of phytochemical compounds, including flavonoids, essential oils, saponins, and alkaloids, which may be useful for medicinal purposes. *Fritillaria thunbergii* Miq. Bulbs are commonly used in traditional Chinese medicine as expectorants and antitussives. In this paper, a feasibility study is presented that examines the use of hyperspectral imaging integrated with convolutional neural networks (CNN) to distinguish twelve (12) Fritillaria varieties (n = 360). The performance of support vector machines (SVM) and partial least squares-discriminant analysis (PLS-DA) was compared with that of convolutional neural network (CNN). Principal component analysis (PCA) was used to assess the presence of cluster trends in the spectral data. To optimize the performance of the models, cross-validation was used. Among all the discriminant models, CNN was the most accurate with 98.88%, 88.89% in training and test sets, followed by PLS-DA and SVM with 92.59%, 81.94% and 99.65%, 79.17%, respectively. The results obtained in the present study revealed that application of HSI in conjunction with the deep learning technique can be used for classification of *Fritillaria thunbergii* varieties rapidly and non-destructively.

## 1. Introduction

The Fritillaria genus consists of several species, all of which have been domesticated in China since 3500 BC. There are three major species within this genus: *Fritillaria thunbergii* (Zhebeimu), Fritillaria chuanbeiiensis (Pingbeimu), and Fritillaria ussuriensis (Chuanbeimu). As well as being a valuable herb, it is also one of the most important economic crops for herb growers. It is estimated that Fritillaria is planted on over 6000 hectares in China. Besides producing over 20,000 tons per year, it also provides farmers with an income of CNY 700 million every year [1]. It has been mentioned in the earliest Chinese herbal monograph “Shen Nong’s Herbal Classic” as a remedy for coughing. In the 2010 version, it remains the same. The Chinese Health Law [2002] No. 51 recognizes all three types of Fritillaria as edible due to their non-toxic nature [2]. Fritillaria is considered to promote lung dispersal, dissolve phlegm, relieve coughing, detoxicate, and dissolve lumps and masses in the chest [1].

Hyperspectral imaging has been incorporated into several research fields using remote sensing. Basically, it involves splitting the electromagnetic spectrum into several bands, thereby providing sufficient spectral resolution while covering a wide range of wavelengths (in this case, hundreds of bands). A hyperspectral image represents the spectrum as a series of images, each representing a narrow band of light, rather than depicting it in two dimensions [3,4].

In addition, this method is non-destructive, rapid, and exhibits a high spectral resolution, thus enabling accurate identification of a variety of chemical compounds. The high spectral resolution allows one to identify unique absorption features in minerals as a result of the interaction between radiation and their crystalline structure [5]. Hyperspectral imaging allows the observation of a variety of wavelengths, including ultraviolet (UV), visible and near-infrared (Vis-NIR), shortwave infrared (SWIR), and longwave infrared (LWIR). Traditional Chinese medicine can be evaluated using hyperspectral imaging [6]. In recent years, spectroscopic techniques and spectral imaging have been widely used to identify agricultural product origins and analyze their quality as rapid, non-destructive testing methods [7,8,9,10,11,12,13,14,15,16,17].

Artificial Intelligence (AI) techniques, such as deep learning (DL), enable machines to acquire knowledge from data autonomously [18]. There are a variety of deep learning models available, but one of the most popular is the convolutional neural network (CNN). A CNN consists of three layers: a convolutional layer, a pooling layer, and a fully connected layer for feature extraction, compression, and classification. Combining several convolutional and pooling layers allows abstract features to be learned more effectively. In the field of computer vision, CNNs have shown remarkable performance in a variety of tasks. As part of hyperspectral image analysis, CNN is used to classify images captured using hyperspectral remote sensing in two and three dimensions [1]. Different CNNs have been developed over the past few years based on specific tasks in spectral analysis, such as single rice seed [17], rice seed varieties [19], hybrid seeds [10], and chrysanthemum varieties [9].

In a limited number of studies, deep learning has been used to identify traditional Chinese medicine. A study is needed to determine whether CNN can discriminate between the varieties of *Fritillaria thunbergii*. The main objective of the study is to examine whether HSI combined with CNN could be used for variety discrimination of *Fritillaria thunbergii* varieties. Specifically, the following objectives were to be achieved: (1) to study the performance of SVM, PLS-DA, and CNN based on the number of training samples, (2) to evaluate the performance of convolutional neural network (CNN) in comparison to support vector machine (SVM) and partial least squares-discriminant analysis (PLS-DA), and (3) to analyze the outcomes of the identification of *Fritillaria thunbergii* varieties according to the best model.

## 2. Materials and Methods

### 2.1. Sample Preparation

The College of Biosystems Engineering and Food Science at Zhejiang University, China, provided 12 different varieties of Fritillaria for the study. The Fritillaria samples were coded as 1, 2, 3, 4, 5, 6, 7, 8, 9, 10, 11, and 12, as presented in (Table 1) for later data processing. Each of the variety had 30 samples; in total, 360 samples were provided. The samples were not subjected to any additional processing. The data set of each variety was divided into a training and testing sets ratio of 4:1 (80:20), respectively.

### 2.2. Hyperspectral Image Acquisition and Correction

The hyperspectral images of *Fritillaria thunbergii* were obtained using a near-infrared HSI system. It consisted of a set of devices that interact: Spectral Imaging Ltd., Oulu, Finland; utilized an imaging spectrograph (ImSpector N17E) that has a spectral range of 874–1734 nm and a high-performance camera (OLES22) that provides a spatial and spectral resolution of 326 × 256 pixels. A stepped motor-driven conveyer belt controlling two 150-Watt tungsten halogen lamps (3900e Light source; Illumination Technologies Inc.; West Elbridge, NY, USA) was used to move the samples. It was determined that 25 cm, 4 ms, and 19.5 mm/s were the appropriate distances between the lens and the conveyor belt to produce clear, non-deformable hyperspectral images. This study acquired hyperspectral images of Fritillaria with 256 spectral channels and a resolution of 5 nm. Using Equation (1), a white and black reference image was used to correct the raw hyperspectral images to reduce the effect of dark currents and determine whether the samples were reflective or not.

(1) $$I_{C}=\frac{I_{raw}-I_{dark}}{I_{white}-I_{dark}}$$

where *I_white_* is the hyperspectral image of a white Teflon tile with nearly 100% reflectance; *I_dark_* is acquired by covering the camera lens with its opaque cap. *I_raw_*, *I_dark_*, *I_dark_* are obtained under the same condition during samples collection.

### 2.3. Pretreatment and Extraction of Spectra

**Figure 1:**
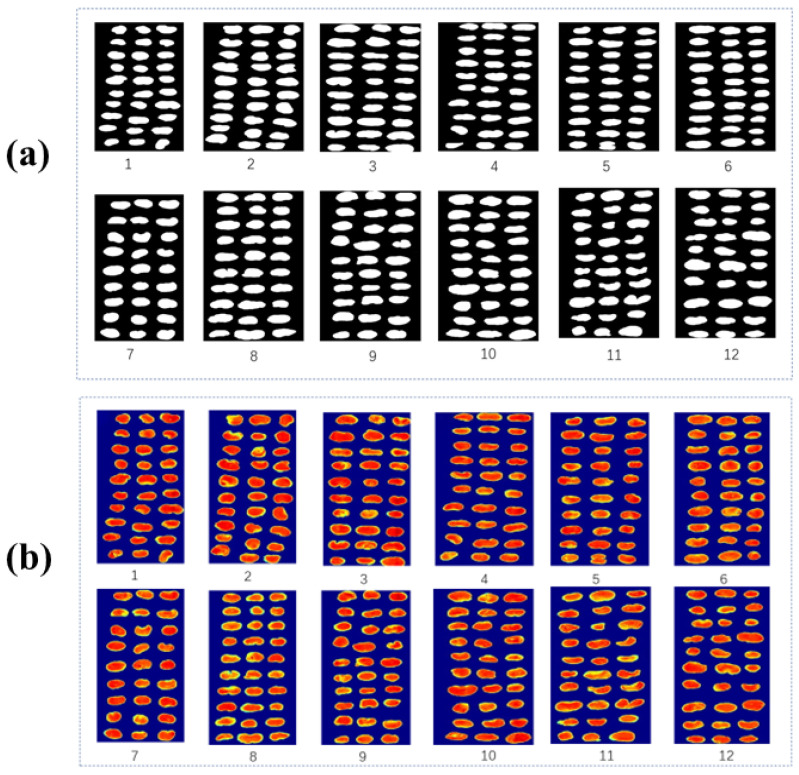
(**a**) Binary image; (**b**) raw colored image.

Before spectral analysis, each Fritillaria sample must be segmented from its black background. For obtaining binary masks, threshold segmentation of an image with maximum contrast between sample regions and the background was performed at 1019 nm using an image with maximum contrast between sample regions and the background. Grayscale images at other wavelengths were also masked with this binary mask to achieve this. Figure 1 illustrates both a binary and raw colored image. Each ROI within each Fritillaria sample was spectrally analyzed for a wavelength range of 974–1634 nm in addition to its ROIs. Instabilities in the hyperspectral imaging system resulted in random noise in the spectral data collected at the beginning and end of the sampling process. In this study, we examined the mid-wavelengths between 875 and 1546 nm. The pixel-wise spectrum was smoothed with a wavelet transform (WT), a decomposition scale of 3, and a primary function of 6. The reduction in spectral noise improved the signal-to-noise ratio. The pixel-wise spectra of each ROI were used to discriminate the different Fritillaria samples.

### 2.4. Software

The Fritillaria samples in the hyperspectral images were cropped from irrelevant backgrounds using ENVI 4.6 (ITT Visual Information Solutions, Boulder, CO, USA). Hyperspectral images were extracted and pre-processed using MATLAB R2018a (The MathWorks, Natick, MA, USA). MATLAB R2018a was also used to implement PCA for pattern recognition between different varieties. Spyder 3.2.6 (Anaconda, Austin, TX, USA) was used to implement Python-based discriminant models, including SVM, PLS-DA, and CNN. Programming was conducted with scikit-learn (http://scikit-learn.org/stable/, accessed on 22 August 2022) and Pytorch (Facebook, Menlo Park, CA, USA). An Intel(R) core (TM) i5-8500 processor with 3.00 GHz and 8G RAM was used as the hardware platform for the execution of all software tools.

### 2.5. Analysis of Chemometrics

#### 2.5.1. CNN

**Figure 2:**
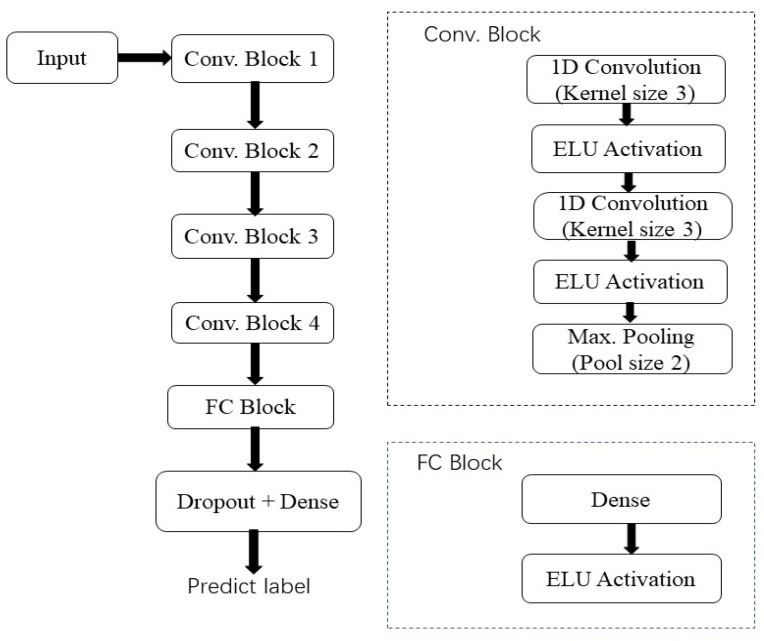
A brief overview of the CNN architecture.

Figure 2 shows an illustration of convolutional neural networks (CNNs). A one-dimensional spectrum input was incorporated into the design of VGGNet [20]. There are similarities between patterns found in spectral curves and those found in images. The peaks and minimums of a spectral curve are analogous to the edges of an image. This network is chosen because of its high performance in image classification and the ease of modification and extension it provides. Figure 2 illustrates the architecture in terms of five main blocks. Two convolutional layers follow a top pooling layer. Deeper blocks have more convolutional filters (starting at 16 and ending at 128). In convolutional layers, there are three kernels, one stride, and one padding. A convolutional algorithm learns local patterns based on its input and local connections. Convolutional layers can be chained together so that deeper layers are connected to a more significant portion of the input data. This results in different layers of learning features based on raw input. The data set of each variety was divided into a training and testing sets ratio of 4:1 (80:20), respectively.

The last block contains a fully connected layer (FC Block). The fully connected layer may learn combinations of features obtained from convolutional layers. It has two layers: dense and dropout layers [21]. The activation function of the original VGGNet architecture was a rectified linear unit (ReLU). The exponential linear unit (ELU) is shown to accelerate learning and outperforms the (ReLU) in some cases [22]. The performance of ELU activation with batch normalization was superior to ReLU activation [23]. Therefore, ELU is implemented as part of the architecture. The following is a description of an ELU function.

(2) $$f(x)=\{\begin{array}{c} x\;\;\;\;\;\;\;\;\;\;\;\;\;\;\;\;\;\;\;\;\;\;\;\;\;\;\;\;\;\;\;\;\;\;\;\;if\;x>0\\ \propto \left(\exp\left(x\right)-1\right)\;\;\;\;\;\;\;if\;x\leq 0 \end{array}$$

As a classification confidence score, values in the range [0, 1] are produced from the CNN output. A classification loss is calculated based on the samples’ confidence scores and their actual labels. As shown in the following equations, softmax and loss function are defined.

(3) $$P_{ij}=\frac{e^{Zij}}{\sum_{k=1}^{K}e^{Zik}}\;for\;j=1,\;K$$

(4) $$\mathrm{Loss}=-\sum_{i}\sum_{j}label_{ij}\log\left(p_{ij}\right)$$

where *Z* represents a CNN input, *i* represents a sample, *j* represents a class, and *K* represents the number of classes.

During CNN training, the data were normalized by dividing the standard deviation by the mean. Before pre-processing the test data, means and standard deviations were calculated on the training data. Initializing the weights of the CNN was carried out by the procedure described in [21]. The Adam algorithm optimized the softmax cross-entropy loss [24]. The following equation showed a gradual decrease in learning rate (ŋ) after training.

(5) $$ŋ=\frac{{ŋ}_{0}}{1+kt}$$

Based on this function, the initial learning rate $\left({ŋ}_{0}\right)$ represents the number of epochs and the decrease in the learning rate represents *k*.

To find the best combination of hyperparameters, a grid search was conducted. A total of 256 batches was generated; dropout ratio was set at 0.5 and ELU at 1.0. To train the CNN, 800 epochs were conducted with the following parameters: h_0_ = 0.0005 and *k* = 0.045.

#### 2.5.2. PLS-DA

This method is considered to be a supervised technique that achieves the maximum level of discrimination between samples in the classification process [25]. The PLS-DA was cross-validated with leave-one-out. The absolute difference between the actual classification number and the predicted value was used to determine discrimination accuracy in both training and test sets. The data set of each variety was divided into a training and testing sets ratio of 4:1, respectively.

#### 2.5.3. SVM

As a pattern recognition method, the Vapnik-Chervonenkis dimension theory and the structural risk minimization principle make SVMs very effective [12,26]. Due to its ability to find a global minimum, SVM differs from neural networks because fewer training samples are required. Radial basis functions (RBFs) are used to construct the kernel function. The data set of each variety was divided into a training and testing sets ratio of 4:1, respectively. A grid-search procedure was used to determine the penalty parameters (c) and kernel function parameters (g).

#### 2.5.4. Discrimination Models Accuracy Evaluation

A well-known F-score was used to assess the discrimination accuracy of the models [27,28,29,30,31,32,33], in comparison to reference classifications, this metric measures the quality of origin discrimination. Specifically, it consists of the precision and recall values that are used to extract information. The precision, recall, and F-score are defined as follows, and their values are presented in Table 2.

(6) $$\mathrm{Precision}=\frac{\mathrm{True}\;\mathrm{positives}}{\mathrm{True}\;\mathrm{positives}+\mathrm{False}\;\mathrm{positives}}$$

(7) $$\mathrm{Recall}=\frac{\mathrm{True}\;\mathrm{positives}}{\mathrm{True}\;\mathrm{positives}+\mathrm{False}\;\mathrm{positives}}$$

(8) $$F-\mathrm{Score}=2\times \frac{\mathrm{Precision}\;\times \;\mathrm{Recall}}{\mathrm{Precision}+\mathrm{Recall}}$$

## 3. Results and Discussion

### 3.1. Spectral Features

**Figure 3:**
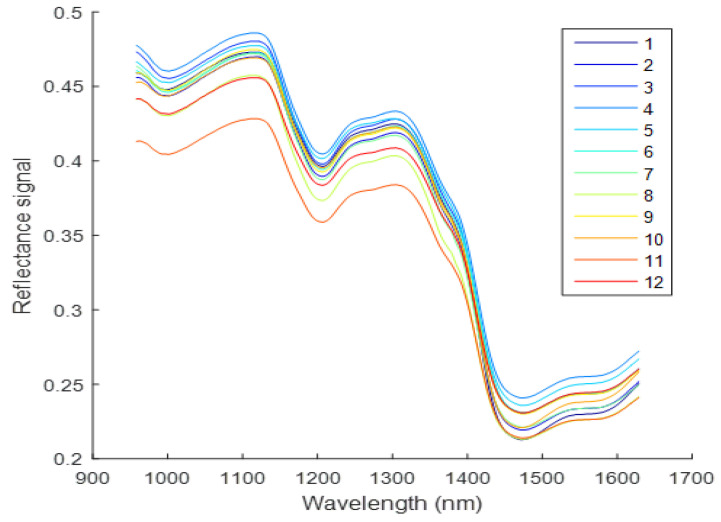
The average spectra of Fritillaria samples of twelve (12) varieties.

Figure 3 illustrates that the valley and peak positions of the average spectra of the twelve (12) varieties were similar. While Fritillaria spectra were generally similar, a few slight differences were observed. These variations in spectrum properties are brought about by the various chemical and biological properties of these twelve (12) kinds. The peaks of spectral curves, at 1100 and 1300 nm, as well as the valleys at 1200 and 1460 nm, could be used to discriminate among Fritillaria varieties. At 1200 nm, the second overtone of C-H stretching is responsible for the two peaks and valleys [16,34]. Furthermore, at 1460 nm, a valley is associated with the first overtone of the O-H stretching [9,34]. The spectral curves of 5, 6, 7, 8, 9, 10, and 11 show a strong overlapping in the range of 950–1200 nm, indicating that these two varieties chemical compositions are similar. The wavelength between 1100 and 1300 nm might be associated with the second overtone of the C-H stretch [35]. Combination bands of C-H vibrations may be responsible for the wavelength between 1300 nm and 1400 nm [36]. The wavelengths between 1400 and 1450 nm might be ascribed to water bands [36]. An overtone of O-H stretch was found to have a wavelength around 1480 nm [37]. CH2 stretching and non-stretching were attributed to the wavelength at 1500 nm [38]. An overtone of the N-H stretch might produce the wavelength between 1500 nm and 1530 nm [39]. An overtone of C-H stretching might account for the wavelength around 1610 nm [40]. The aromatic C-H band was attributed to the wavelength around 1630 nm [11]. These wavelengths carrying the category information are closely related to the constituent differences in chemical composition of different Fritillaria variety.

### 3.2. PCA

Spectral data analysis is commonly performed using the principal component analysis (PCA). Using PCA, principal components (PCs) were determined from linear combinations of original variables. Depending on the interpretation of the variation, PCs are positioned orthogonally. First, PC examined nearly all variations, followed by second, third, etc. Generally, the first few PCs analyzed most variations [30,41]. PCAs are often used as a qualitative method of spectral analysis. This study used PCA to compare twelve (12) varieties of Fritillaria. During the PCA analysis, hyperspectral images of each variety of the testing set were randomly selected. Approximately 78.77% and 17.35% of the information in the original spectral data is reflected in the first and second PC, respectively.

**Figure 4:**
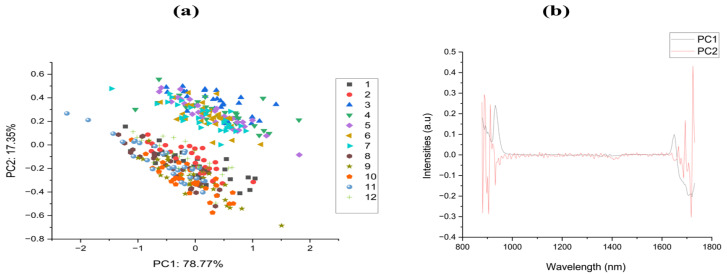
(**a**) 2D-distribution of the twelve (12) individual varieties (**b**) Loadings.

Conversely, the first two PCs accounted for 96.12% of the variance. Based on this analysis, the two peak PCs contain virtually all spectral information for the various spectrum regions. Figure 3 shows the mean spectra of 12 varieties of Fritillaria. It was observed that the reflectance curves of Fritillaria resembled those of Fritillaria in [6,42]. As shown in Figure 4, there is little overlap between the twelve varieties. There appears to be a disconnection between the varieties. There was a rough separation of the Fritillaria samples. According to the PCA analysis, different varieties have different chemical compositions. Although the cluster trend could be observed in two dimensions, no distinction could be made between the samples. Consequently, discriminant algorithms were employed in this study [30,43,44].

### 3.3. CNN

Using a CNN as the discriminant model, Fritillaria samples were correctly classified. SVM and PLS-DA were introduced as contrast methods. The data set of each variety was divided into a training and testing sets ratio of 4:1, respectively. Spectral imaging requires machine learning methods to interpret the spectral data derived from various spectroscopy techniques. Artificial intelligence is focused on deep learning, and convolutional neural networks are among the most popular deep learning models. Two-dimensional images are typically analyzed using deep learning methods [17]. This study found that CNN can perform well when applied to one-dimensional spectra. CNN model showed improved performance over the SVM and PLS-DA models. As shown in this study, a new method for analyzing spectral data can be developed using CNN, which provides new methods for handling spectral data. Varieties of Fritillaria may differ greatly in chemical composition due to environmental influences, cultivation management, and other factors. Qiu et al. [17] analyzed whether hyperspectral imaging and convolutional neural networks can be used to distinguish rice seed varieties. Four rice seeds were photographed using hyperspectral imaging techniques in the 380–1030- and 874–1734-nanometer spectral regions. Generally, CNN models outperformed SVMs and KNNs, showing CNN’s effectiveness in analyzing spectral data. It was shown in this study that CNN had positive outcomes when used for the analysis of spectral data. Acquarelli et al. [45] suggested using a CNN structure to analyze data from vibrational spectroscopy, as comparison methods, PLS-DA, logistic regression, and KNN were employed. The CNN model demonstrated a good outcome. While CNN consistently outperformed other models, it was not always the best option. Liu et al. [46] classified pre-processed and not pre-processed Raman spectra using a CNN architecture. CNN performed better than models based on KNN, SVM, gradient boosting, random forest, and correlation analysis. In addition to the results presented in this paper, this study concludes that CNN can be used to analyze one-dimensional spectral data. The number of training samples was also examined to ascertain whether it affected the results. In general, as the number of training samples increases, the performance of machine learning methods increases. Models that have been trained cannot perform well on tests due to a lack of training samples. In view of the redundancy of information within the training samples, once a certain point is reached, performance may no longer be significant. Additionally, collecting samples may take considerable time [17]. It is, therefore, important to strike a balance between model performance and cost. With increased training samples, CNN outperformed SVM and PLS-DA models. A deep learning method may be able to learn features automatically, and more samples may enable a deeper exploration of potential feature combinations. In practical applications, models should be developed that are capable of identifying more Fritillaria varieties. Keeping a hold-out set of test data and gradually collecting samples for training so that there is no significant change in the test accuracy is essential if high model performance is achieved reasonably.

### 3.4. PLS-DA

**Figure 5:**
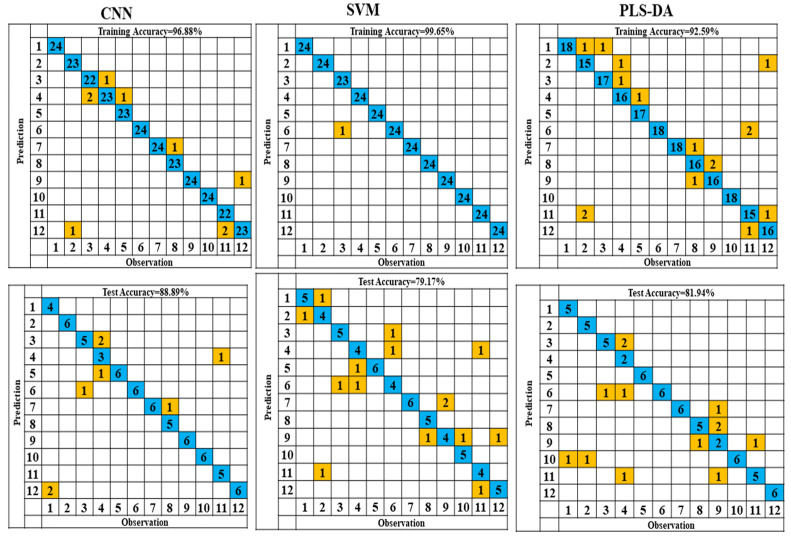
Confusion matrices of the different models.

Based on the confusion matrices, the results of the various models for the 12 varieties of Fritillaria are illustrated in Figure 5. Different varieties of Fritillaria produce different results when compared with different models. As the number of Fritillaria varieties increased, the performance of the PLS-DA model decreased. In linear classification, PLS-DA is an efficient approach [47]. Regarding the classification of seeds, neural networks and nonlinear models, such as support vector machines, outperformed linear models [17,48,49,50]. Based on a CNN discriminant model, 96.88% and 88.89% recognition accuracy were achieved in training and test sets, respectively, which were superior to the classification accuracy achieved for twelve (12) varieties of *Fritillaria thunbergii* by SVM and PLS-DA. The CNN effectively extracted features from the spectral data because it contained the most in-depth information. Many deep features were present in the spectral data, which were more easily extracted with a CNN. The deep architecture of deep learning models allows the extraction of more abstract and non-changing features from the data, resulting in a higher level of performance than traditional shallow classifiers [51]. Figure 5 illustrates how CNN and PLS-DA discriminant analysis models can maintain a relatively high level of performance compared to SVM.

### 3.5. SVM

**Figure 6:**
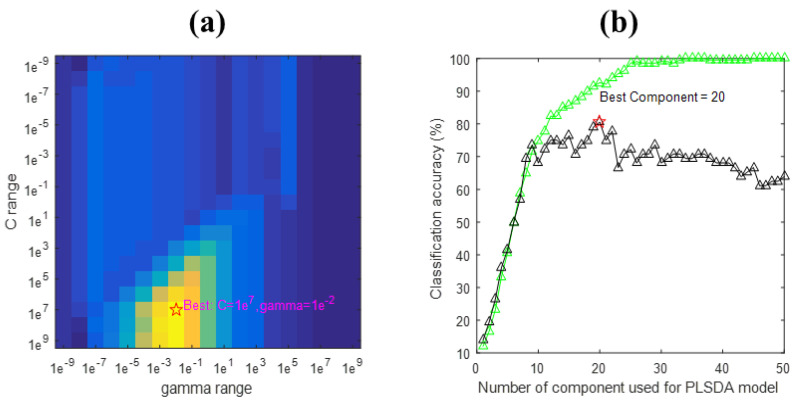
(**a**) The grid-search result for the optimization of the radial basis function support vector machine classifier (RBF-SVC0 model). The best combination of the RBF-SVC parameters is marked by ‘*’. (**b**) PLS-DA: Best component = 20.4. The best combination of the RBF-SVC parameters is marked by the red five-pointed star. The Green line is the training accuracy and The Black line is for validation.

The SVM algorithm is a nonlinear machine learning algorithm that uses nonlinear hyperplanes to classify complex data sets [26]. As the number of Fritillaria varieties increased, the discriminant performance of the SVM decreased, and the training and test sets were recognized accurately with an accuracy of 99.65% and 79.17%, respectively. Zhao et al. classified maize seeds using a radial basis function neural network, with calibration and prediction accuracy of 98.03% and 93.26%, respectively [13]. There was a problem of over-fitting in the SVM-based discriminant analysis model. It has been shown that discriminant models undergo overfitting when used with NIR hyperspectral imaging data [12,17,52]. A grid-search procedure was used to determine the penalty parameters (c), kernel function parameters (g), and best component in Figure 6. By using dropouts and batch normalization, discriminant models can be improved [10]. Deep learning algorithms provide superior models for discriminant analysis [17,20,52]. Comparing CNN with SVM and PLS-DA models, the CNN model performed significantly better when the number of Fritillaria varieties was increased. In addition, the discriminant model based on CNN was optimized to minimize the problem of overfitting. This allows the CNN to be used for the classification of *Fritillaria thunbergii* varieties using hyperspectral imaging technology. It is imperative that additional Fritillaria varieties be collected in order to develop an instrument for identifying Fritillaria varieties. Furthermore, a comprehensive research effort will be required in the future to assess the quality of Fritillaria.

## 4. Conclusions

The capability of hyperspectral imaging technology to discriminate was demonstrated using machine learning algorithms. The classification of Fritillaria varieties was successfully achieved by three algorithms. The accuracy of each model was optimized through cross-validation (CV), which was determined using the highest classification rate for each model. CNN model showed improved performance over the SVM and PLS-DA models, with F-scores of 89.38%, 79.63%, and 82.63%, respectively. There has been little research examining deep learning algorithms for the classification of traditional Chinese medicine using HSI, making this study an important contribution to the field. As a result of the investigation, some conclusions have been drawn. Developing more robust origin models capable of detecting regional and temporal variations is necessary in the future. A large data set representing a wide range of variability (such as geographical origin, harvest period, and harvest year) should be conducted. The results obtained in the present study revealed that application of HSI in conjunction with the deep learning technique can be used for classification of *Fritillaria thunbergii* varieties rapidly and non-destructively.

## Figures and Tables

**Table 1:** Information on different varieties of Fritillaria samples.

ID.	Variety	State	Origin	Supplier
1	TongRenTang	Flake	Zhejiang, China	Tongrentang (Sichuan) Health Pharmaceutical Co., Ltd.
2	MoYuan	Flake	Zhejiang, China	Anguo MedicineSource Trading Co., Ltd.
3	NiuEnTang	Flake	Zhejiang, China	Hebei NiuEntang Electronic Commerce Co., Ltd.
4	QiGuiTang	Flake	Zhejiang, China	Hebei Lingkang Trading Co., Ltd.
5	ZeXinTang	Flake	Zhejiang, China	Bozhou ZeXinTang Pharmaceutical Co., Ltd.
6	JiaQiTang	Flake	Zhejiang, China	Anguo Guangsheng Trading Co., Ltd.
7	FuXiTang	Flake	Zhejiang, China	Sichuan Haorui Gallium Biotechnology Co., Ltd. (Sichuan)
8	ZangXiTang	Flake	Zhejiang, China	Sichuan Zangxitang Biotechnology Co., Ltd.
9	NanBeiHang	Flake	Zhejiang, China	Guangzhou NanBeiHang Chinese Medicine Herb Co., Ltd.
10	ShenYue	Flake	Zhejiang, China	Tonghua Sanbao Ginseng Antler Trading Co., Ltd.
11	KangMei	Flake	Zhejiang, China	Kangmei Pharmaceutical Co., Ltd. (Guangdong)
12	YiLing	Flake	Zhejiang, China	Shijiazhuang Yiling Herbal Pieces Co., Ltd.

**Table 2:** Precision, Recall, and F-Score values of the three models.

Models	Data Set	Precision (%)	Recall (%)	F-Score
CNN	Training	0.9705	0.9688	0.9697
	Testing	0.8988	0.8889	0.8938
SVM	Training	0.9967	0.9965	0.9965
	Testing	0.8010	0.7917	0.7963
PLS-DA	Training	0.9267	0.9259	0.9263
	Testing	0.8333	0.8194	0.8263

## Data Availability

The data presented in this study are available on request from the corresponding author. The data are not publicly available due to privacy.
